# Comparison of nitrogen transformation dynamics in non-irradiated and irradiated alfalfa and red clover during ensiling

**DOI:** 10.5713/ajas.18.0695

**Published:** 2019-03-07

**Authors:** Zhihao Dong, Junfeng Li, Lei Chen, Xianjun Yuan, Tao Shao

**Affiliations:** 1 Institute of Ensiling and Processing of Grass, College of Agro-grassland Science, Nanjing Agricultural University, Nanjing 210095, China

**Keywords:** Alfalfa, Ensiling, Irradiation, Nitrogen Transformation, Red Clover

## Abstract

**Objective:**

To study the contribution of plant enzyme and microbial activities on protein degradation in silage, this study evaluated the nitrogen transformation dynamics during ensiling of non- and irradiated alfalfa (*Medicago sativa* L.) and red clover (*Trifolium pratense* L.).

**Methods:**

Alfalfa and red clover silages were prepared and equally divided into two groups. One group was exposed to γ-irradiation at a recommended dosage (25 Gky). Therefore, four types of silages were produced: i) non-irradiated alfalfa silage; ii) irradiated alfalfa silage; iii) non-irradiated red clover silage; and iv) irradiated red clover silage. These silages were opened for fermentation quality and nitrogen components analyses after 1, 4, 8, and 30 days, respectively.

**Results:**

The γ-irradiation successfully suppressed microbial activity, indicated by high pH and no apparent increases in fermentation end products in irradiated silages. All nitrogen components, except for peptide-N, increased throughout the ensiling process. Proteolysis less occurred in red clover silages compared with alfalfa silages, indicated by smaller (p<0.05) increment in peptide-N and free amino acid N (FAA-N) during early stage of ensiling. The γ-irradiation treatment increased (p<0.05) peptide-N and FAA-N in alfalfa silage at day 1, whereas not in red clover silage; these two nitrogen components were higher (p<0.05) between day 4 and day 30 in non-irradiated silages than the irradiated silages. The ammonia nitrogen and non-protein nitrogen were highest in non-irradiated alfalfa silage and lowest in irradiated red clover silage after ensiling.

**Conclusion:**

The result of this study indicate that red clover and alfalfa are two forages varying in their nitrogen transformation patterns, especially during early stages of ensiling. Microbial activity plays a certain role in the proteolysis and seems little affected by the presence of polyphenol oxidase in red clover compared with alfalfaa.

## INTRODUCTION

Ensiling is a common method of preserving forage crops after harvest. However, ensiling reduces protein quality compared with the fresh forage due to the occurrence of proteolysis during ensiling. For some ensiled forages, such as alfalfa (*Medicago sativa* L.), proteolytic losses can be especially high, with degradation of 44% to 87% of the forage protein [1]. The forage protein can be converted into non-protein N (ammonia, amino acids, and small peptides), which is poorly utilized by ruminant animals, resulting in not only high economic losses to farmers but also nitrogen burdens upon the environment [2].

Various factors can influence the rate and extent of proteolysis during ensiling [3]. It has generally been accepted that a rapid pH decline plays a crucial role in minimizing protein degradation during ensiling [4]. However, other factors, such as forage species, could also strongly affect the extent of proteolysis during storage. In comparison to alfalfa, red clover (*Trifolium pratense* L.) shows lower extent of proteolysis, with only 7% to 40% of its protein being degraded during ensiling [5]. The main factor leading to this difference is speculated to relate the presence of natural protein-protecting mechanism in red clover, i.e. the activity of polyphenol oxidase (PPO) which can catalyze the oxidation of endogenous phenols to quinones. These quinones react with nucleophilic sites on cellular proteins, forming protein-phenol complexes to resist proteolysis or/and inactivate proteases [6]. However, although the fact that PPO reduces proteolysis is well understood [7,8], it is unclear how nitrogen components change during ensiling of alfalfa and red clover. Furthermore, it has been suggested that the depressed proteolysis in red clover results from the deactivation of plant proteases due to the action of PPO [9,10]. However, protein degradation in silo is a process in which both plant and microbial proteases are involved [11]. It has not been understood whether microbial activity can be influenced by the presence of PPO.

Irradiation treatment is an approach often used to differentiate the effects of microbial activity from those of plant enzymes. Many studies have shown that irradiation treatment can effectively sterilize forage while having little effect on plant enzymes [11,12]. The objectives of this study were to characterize the dynamics of nitrogen transformations in ensiled alfalfa and red clover, and to investigate the effects of legume species and microbial activities on nitrogen components of silage through γ-irradiation treatment.

## MATERIALS AND METHODS

### Ensiling materials

Alfalfa and red clover were cultivated in an experimental field of Nanjing Agricultural University, Nanjing, Jiangsu, China (N31° 14″, E118° 22″). Two forages had similar fertilizer management and were harvested at late bud to early bloom on September 8, 2017. The stubble was 10 cm above ground level. Fresh forages were transported to laboratory and wilted for 1 day on a plastic sheet. After that, wilted forages were chopped into a theoretical cutting length of 1 to 2 cm using a forage chopper (F5, XiangLong, Co., Ltd., Linyi, China).

### Experiment design and silage preparation

Chopped alfalfa and red clover (approximately 780 g) were packed into silos (polyethylene bottles, 1 L capacity), which were weighed and sealed with plastic caps and adhesive tapes. Silos containing alfalfa or red clover were respectively divided into two groups. One group was sterilized by exposure to γ-irradiation at 25 kGy for 2 h at room temperature, after filling. Irradiation at this dosage has been previously shown to effectively suppress microbial activity in alfalfa silage [11]. Therefore, four silage treatments were prepared: i) non-irradiated alfalfa silage; ii) irradiated alfalfa silage; iii) non-irradiated red clover silage; and iv) irradiated red clover silage. These silos were stored at ambient temperature (18°C to 22°C) in the laboratory. For each treatment, sixteen silos were prepared and four silos opened after 1, 4, 8, and 30 days of ensiling. At silo opening, the silages were transferred to a plastic box for homogeneous mixing, after which subsamples were taken for chemical and microbial analyses.

### Chemical and microbial analyses

The buffering capacity was determined with the fresh forages by the method of Playne and Mcdonald [13]. Approximately 0.5 kg of fresh forage and silage samples were oven-dried at 60°C for 48 h to determine dry matter concentration. The dried samples were ground to pass a 1 mm screen in a laboratory knife mill (FW100, Taisite Instrument Co., Ltd., Tianjin, China), and used for analyses of total nitrogen (TN). The TN was determined with the method of Association of Official Analytical Chemists [14]. Crude protein content was calculated by a multiplication factor (6.25) from TN.

Thirty five grams of fresh forages and silages were extracted in 70 mL of deionized water at 4°C for 24 h to obtain the cold extract, which was used for determination of silage fermentation parameters according to the procedures of Chen et al [15]. The pH of the water extract was measured with an electrode pH meter (HANNA pH 211, Hanna Instruments, Padua, Italy). The contents of lactic acid (LA), acetic acid (AA), propionic acid, butyric acid, and ethanol were quantified with the filtrates using a high-performance liquid chromatography (Carbomix H-NP5 column, 55°C, 2.5 mM H_2_SO_4_, 0.5 mL/min) as described as Ding et al [11]. A 10-mL aliquot of 25% (w/v) trichloroacetic acid (TCA) was added to 40 mL extract and allowed to stand at room temperature for 1 h to precipitate the protein. The solution was then centrifuged at 4°C, 18,000×g for 15 min, and the supernatant was analyzed for ammonia nitrogen (NH_3_-N) and free amino acid nitrogen (FAA-N). Peptide-N concentration was determined by the increase in FAA-N in the TCA supernatant after digesting with 6 N HCl for 21 h at 105°C, under an N2 atmosphere [16]. Another 10-mL aliquot of TCA-treated supernatant was used for the measurement of non-protein N by the method as described by Licitra et al [17].

To test the effect of γ-irradiation treatment on microbial composition and PPO activity, approximately 0.5 kg of the wilted forages were sampled before and after exposing to γ-irradiation. The lactic acid bacteria (LAB) was counted on de Man, Rogosa and Sharpe agar medium, incubated in an anaerobic incubator at 37°C for 3 days. Yeasts and aerobic bacteria were counted after incubation at 37°C for 2 days. A 0.5-g aliquot was sampled before and after γ-irradiation for PPO activity determination according to the procedures of Winters et al [7]. The Bio-Gel P6DG (Biorad Ltd, Hemel Hempsted, UK) column was used to desalt crude enzyme extract and one unit of PPO activity was determined as a change of 0.01 in absorbance per min. The PPO activity was expressed per gram of fresh matter of the sample (U/g FM).

### Statistical analysis

All results were analyzed using the MIXED procedure of SAS (SAS Enterprise Guide 6, SAS Institute Inc., Cary, NC, USA). Fermentation parameters and nitrogen components of non-irradiated and irradiated alfalfa and red clover were compared using the following model: *Y_ijk_* = *μ*+*S_i_*+*A_j_*+*T_k_*+*S_i_*×*A_j_*+*S_i_*×*T_k_*+*A_j_*×*T_k_*+*S_i_*×*A_j_*×*T_k_*+*ɛ* with *S_i_* the effect of ensiling day; *A_j_* fixed effect of forage species; *T_k_* the fixed effect of γ-irradiation treatment. Interactions between the effects were tested in the same model. Tukey’s multiple comparison was used for the means separation. Significant differences were declared when p<0.05.

## RESULTS

### Characteristics of the forages

The characteristics of fresh alfalfa and red clover are shown in Table 1. The chemical compositions did not differ between the two forages.

### Effect of γ-irradiation treatment on PPO activity and microbial composition

The PPO activity and microbial composition of the two forages before and after exposure to γ-irradiation are presented in Table 2. As expected, PPO activity was not detected in alfalfa, whereas was highly active in red clover (15.6 U/g FM). Treating γ-ray radiation did not influence (p>0.05) red clover PPO activity. The PPO activity of irradiated red clover was 14.1 U/g FM. The microbial populations including LAB, yeast and aerobic bacteria were suppressed (p<0.05) after γ-ray radiation treatment.

### Effect of γ-irradiation treatment on fermentation parameters

Since one of the objectives was to assess the role of microbial activity in silos, γ-irradiation was used to inhibit microbial growth. To determine whether microbial activity was suppressed during ensiling with the γ-ray radiation treatment, fermentation parameters were examined (Table 3). The fermentation parameters of irradiated silages differed from those of non-irradiated silages. The pH of irradiated silage remained higher than that of control silages and no apparent increases in fermentation end products (LA, AA, and ethanol) during ensiling. In contrast, the non-irradiated silages demonstrated stronger fermentation, with greater declined pH and greater accumulation of fermentation end products throughout the ensiling process.

### Nitrogen transformation dynamics in the silage

The nitrogen components concentrations including peptide-N, FAA-N, NH_3_-N and non-protein nitrogen (NPN) (all on a TN basis) at each ensiling interval are shown in Table 4. In the four types of silages all nitrogen components concentrations increased with duration of ensiling except for peptide-N, which first increased at the early stage and decreased later.

Forage species affected peptides N concentration in silage, with alfalfa silages showing greater (p<0.05) peptide-N compared with red clover silages at each time frame of ensiling. At day 1 the γ-ray radiation treatment increased (p<0.05) peptide-N, whereas not in red clover silage.

Similar as peptide-N, larger increases of FAA-N in alfalfa silages were observed before day 1 compared with red clover silages. Between day 4 and day 30, alfalfa and red clover silages treated with γ-ray radiation exhibited lower FAA-N compared with their control counterparts (p<0.05).

The NH_3_-N concentrations were affected by forage species and γ-ray radiation treatment. Red clover silage showed lower NH_3_-N compared with alfalfa silage throughout the whole ensiling process (p<0.05). Treatment of γ-ray radiation reduced NH_3_-N in alfalfa and red clover silages (p<0.05). At the end of ensiling, NH_3_-N was highest in control alfalfa silage and lowest in irradiated red clover silage.

The NPN concentration increased by 5.50 times in alfalfa silage and 4.85 times in red clover silage to the end of tested period. The most rapid increments in NPN were observed during the first day. For the thirty-day silages, γ-ray radiation treatment reduced NPN concentration by 6.74% in alfalfa and by 21.8% in red clover.

## DISCUSSION

### The γ-irradiation inhibited microbial activity and showed little damage to plant enzymes

To separate the effects of plant and microbial activities on major nitrogen components in silage, several methods have been previously devised. These include the production of microbe-free grass grown under asceptic condition [18], the use of antimicrobial agents such as toluene, chloroform and various antibiotics [19], and the use of γ-irradiation [20]. Of these, treatment of γ-irradiation might be most advisable. Despite some bacterial spores, such as those of clostridia, being very resistant to irradiation treatment, the irradiation can easily kill most microorganisms and, if at proper dose, shows no or minor damage to plant enzymes [21]. In the present experiment, the 25 kGy, as suggested by Ding et al [11], indicated effective suppression of microbial activity. Butyric acid was undetected in all silages, regardless of irradiation (data not shown). This was likely resulted from a low population of clostridia on the fresh forages. The PPO activity in fresh red clover was not significantly altered after exposing to γ-irradiation, partly reflecting that the plant enzyme activity was not affected by the γ-irradiation treatment.

Overall, the γ-irradiation treatment effectively suppressed microbial activity and showed little effect on plant enzyme activity. The treatment in this study provided a satisfactory mode which would allow the investigation of effects of plant and microbial activities on nitrogen components of alfalfa and red clover silages, despite causing some small changes in chemical composition of the forages.

### Nitrogen transformation dynamics in the silage

Degradation of plant protein during ensiling is inevitable and results in the transformation of true protein into NPN of low poor nutritive value [22]. Two known steps are involved in the process of protein transformation: Firstly, peptides bond hydrolysis (proteolysis) occurs, resulting in the formation of free amino acid and peptides. Secondly, amino acids are further degraded into a range of end products, including ammonia, organic acids, and amines. Plant protease is mainly responsible for transformation of the true protein to free amino acids and peptides, and further amino acid metabolism is the result of microbial protease [23].

As a direct indicator of hydrolysis, peptides are formed by proteolysis and further degraded by deamination and decarboxylation activities by microorganisms. Compared with alfalfa silage, red clover silage, regardless of irradiation, exhibited lower peptide-N during early stages of ensiling, suggesting that less proteolysis occurred. This lower extent of protein breakdown was supposed to result from deactivation of plant proteases due to the action of PPO [10]. It has been proposed that the mechanism of PPO-reduced proteolysis is related with the generation of o-quinones that react with nucleophilic sites on cellular proteins, reforming as a phenol covalently bound to protein. Proteases including carboxypeptidase, aminopeptidase and acid proteinase activities in red clover were thus lower compared with those in non-PPO containing forages, such as alfalfa [24]. Also, a reduction in general protein solubility through protein-phenol binding has been shown to decrease the accessibility of forage proteins to proteases, which were demonstrated to reduce protein breakdown during ensiling [25]. The γ-irradiation treatment increased peptide-N in alfalfa silage at the initial stage of ensiling, which might be attributed to the absence of microbial fermentation, resulting in a favorable pH for plant protease, thereby increasing the extent of proteolysis. However, the increase of peptide-N was not observed in the irradiated red clover silage. This could be explained by the low plant protease activity that was unable to cause a larger extent of proteolysis even under a favorable silage pH. Several studies have previously shown that peptide-N first increased and then decreased thereafter at later stages of ensiling [22,24]. They ascribed the decreased peptide-N to the deamination and decarboxylation activities by microorganisms at later stages of ensiling. In the present study, silages treated with γ-irradiation also showed decreases in peptide-N after the highest point. This was particularly evident in irradiated alfalfa silage, indicating that plant enzyme and microbial activities both contributed to the further degradation of peptides.

The dynamics of peptide and FAA during the ensiling process are highly related, since they are both hydrolysis products of true protein [24]. Similar as peptide, FAA can be consumed by microorganisms as substrates for deamination to produce NH_3_; its content is largely dependent on the relative activity between hydrolysis and deamination [22]. Compared with their irradiated counterparts, non-irradiated alfalfa and red clover silages showed higher FAA-N between day 4 and day 30. The production of more FAA-N might be attributed to the increasing microbial protease activity which replaced the dominant activity of plant protease at later stages of ensiling. This is in agreement with previous studies [26,27].

As one product of further amino acids breakdown, ammonia is formed from microbial activity, rather than from plant enzymes [26]. Ammonia can be an indicator to estimate the microbial activity in silage. In this study, red clover silage showed lower ammonia compared with alfalfa silage during the whole ensiling process. This was likely because weak proteolysis in red clover resulted in less FAA substrates available for microbial deamination [28]. Due to the suppressed microbial activity, the γ-irradiated silages also exhibited lower ammonia than their non-irradiated counterparts. There was no interaction between forage species and γ-irradiation on ammonia concentration, reflecting that the effect of the presence of PPO on microbial activity during ensiling was minor. This could be explained by the fact that microbial activity became abundant at stages where PPO had already lost activity.

Regardless of forage species, the most rapid increments in NPN were observed during the first day, suggesting that most of the protein breakdown took place within the first day of ensiling. This is consistent with Guo et al [26], who reported that most rapid proteolysis in alfalfa occurred during the first 24 hours of ensiling. Benefited by PPO protein protection, protein breakdown occurred less in red clover silages compared with that in alfalfa silages. At the end of ensiling, the small difference in NPN concentration between non-irradiated and irradiated silages (alfalfa and red clover), revealed the subordinate role of microbial protease in the whole proteolysis process during ensiling, consistent with previous reports [11].

## CONCLUSION

This study was able to demonstrate the separate contribution of legume species effect (plant enzyme) from microbial activity throughout ensiling stages using γ-irradiation treatment. The results of this study indicate that red clover and alfalfa are two forages varying in their nitrogen transformation patterns, especially during early stages of ensiling. The microbial activity plays a certain role in proteolysis and seems little affected by the presence of PPO in red clover compared with alfalfa.

## Figures and Tables

**Table 1:** The characteristics of fresh alfalfa and red clover

Items	Alfalfa	Red clover	SEM	p-value
Chemical composition				
DM (% FM)	26.8	27.1	1.39	0.055
Crude protein (g/kg DM)	277	271	6.47	0.379
TN (g/kg DM)	44.3	43.3	1.03	0.379
Buffer capacity (mEq kg/DM)	442	385	7.51	0.105

DM, dry matter; FM, fresh matter; TN, total nitrogen; SEM, standard error of means (n = 4).

**Table 2:** Effect of f γ-irradiation treatment on polyphenol oxidase activity and microbial composition

Forage species	Treatment	PPO activity (U/g FM)	Microbial composition (cfu/g FM)		

			LAB	Yeast	Aerobic bacteria
Alfalfa	Control	ND	6.27	4.66	5.26
	γ-Ray	ND	ND	ND	ND
Red clover	Control	15.6	6.07	4.72	5.58
	γ-Ray	14.1	ND	ND	ND
SEM		0.25	0.04	0.06	0.09
p-value					
Forage species		<0.001	0.228	0.581	0.182
γ-Ray		0.241	<0.001	<0.001	<0.001
Species×γ-ray		0.180	0.228	0.581	0.581

PPO, polyphenol oxidase; FM, fresh matter; cfu, colony-forming units; LAB, lactic acid bacteria; SEM, standard error of means (n = 4); ND, non-detectable.

**Table 3:** The fermentation parameters of alfalfa and red clover treated with or without γ-ray radiation

Stage	Forage species	DM (%) FM		pH		LA (g/kg DM)		AA (g/kg DM)		Ethanol (g/kg DM)	
				
		Control	γ-Ray	Control	γ-Ray	Control	γ-Ray	Control	γ-Ray	Control	γ-Ray
One-days silage	Alfalfa	26.0	26.0	5.56^A^^a^	6.28^B^^b^	34.3^B^^b^	12.6^a^	15.9	13.1	33.3^B^	29.0^B^
	Red clover	26.7	27.5	6.07^B^	5.90^A^	15.1^A^^b^	11.7^a^	12.3	11.2	20.1^A^	17.8^A^
Four-days silage	Alfalfa	23.5	24.1	5.78^a^	6.28^b^	42.9^B^^b^	13.9^a^	17.4	13.7	31.2^B^	34.1^B^
	Red clover	24.5	24.8	5.98	5.95	21.5^A^^b^	15.7^a^	15.5	13.6	20.8^A^	20.8^A^
Eight-days silage	Alfalfa	23.8	24.8	4.80^a^	5.85^b^	57.8^b^	13.1^a^	20.2^b^	13.0^a^	34.3	31.3^B^
	Red clover	24.9	24.5	5.07^a^	5.85^b^	50.2^b^	12.5^a^	17.3^b^	12.0^a^	28.1^b^	19.7^A^^a^
Thirty-days silage	Alfalfa	23.8	25.2	5.35^a^	5.93^b^	38.3^A^^b^	13.2^a^	46.1^B^^b^	13.8^a^	38.7^B^^b^	31.0^B^^a^
	Red clover	23.2	25.9	4.94^a^	5.56^b^	57.7^B^^b^	12.5^a^	29.2^A^^b^	12.1^a^	27.8^A^^b^	19.5^A^^a^
SEM		0.455		0.046		0.627		0.269		0.581	
p-value											
Forage species		0.861		0.475		0.007		<0.001		<0.001	
γ-Ray radiation		0.963		<0.001		<0.001		<0.001		0.002	
Forage species×γ-ray radiation		0.467		0.028		0.008		<0.001		0.474	

DM, dry matter; FM, fresh matter; LA, lactic acid; AA, acetic acid; SEM, standard error of means (n = 4).

A,B At each ensiling interval values in the same column with different capital letter differ significantly between alfalfa and red clover.

a,b Values in the same row with different lowercase letter differ significantly between treatment and control.

**Table 4:** The nitrogen components of alfalfa and red clover silages treated with or without γ-ray radiation

Stage	Forage species	Peptide-N (g/kg TN)		FAA-N (g/kg TN)		NH_3_-N (g/kg TN)		NPN (g/kg TN)	
			
		Control	γ-Ray	Control	γ-Ray	Control	γ-Ray	Control	γ-Ray
Fresh forage	Alfalfa	56.1^B^	56.1^B^	15.8^B^	15.8^B^	0	0	80.1^B^	80.1^B^
	Red clover	41.6^A^	41.6^A^	7.16^A^	7.16^A^	0	0	47.9^A^	47.9^A^
One-days silage	Alfalfa	91.6^a^	297^B^^b^	112^B^^a^	224^B^^b^	26.8^B^^b^	11.8^a^	293^B^^a^	447^B^^b^
	Red clover	103	95.1^A^	41.4^A^^b^	18.8^A^^a^	12.1^A^	8.60	198^A^^b^	153^A^^a^
Four-days silage	Alfalfa	311^B^^b^	210^B^^a^	149^B^	159^B^	26.8	18.6	479^B^^b^	395^B^^a^
	Red clover	83.9^A^^a^	107^A^^b^	49.2^A^	45.5^A^	24.1^b^	14.2^a^	206^A^	211^A^
Eight-days silage	Alfalfa	247^B^^b^	221^B^^a^	297^B^^b^	213^B^^a^	58.4^B^^b^	24.4^B^^a^	511^B^^b^	420^B^^a^
	Red clover	148^A^^b^	106^A^^a^	77.9^A^^b^	37.4^A^^a^	33.8^A^^b^	16.2^A^^a^	286^A^^b^	201^A^^a^
Thirty-days silage	Alfalfa	259^B^^b^	236^B^^a^	297^B^	321^B^	78.0^B^^b^	35.4^B^^a^	519^B^	484^B^
	Red clover	71.5^A^	80.6^A^	56.6^A^	60.1^A^	48.2^A^^b^	22.1^A^^a^	280^A^^b^	219^A^^a^
SEM		4.76		1.99		1.77		5.91	
p-value									
Forage species		<0.001		<0.001		<0.001		<0.001	
γ-Ray radiation		0.017		0.004		0.021		<0.001	
Forage species×γ-ray radiation		<0.001		<0.001		0.203		<0.001	

TN, total nitrogen; FAA-N, free amino acid N; NH_3_-N, ammonia N; NPN, non-protein N; SEM, standard error of means (n = 4).

A,B At each ensiling interval values in the same column with different capital letter differ significantly between alfalfa and red clover.

a,b Values in the same row with different lowercase letter differ significantly between treatment and control.
